# Sex differences in the relationship between dietary pattern adherence and cognitive function among older adults: findings from the NuAge study

**DOI:** 10.1186/s12937-020-00575-3

**Published:** 2020-06-20

**Authors:** Danielle D’Amico, Matthew D. Parrott, Carol E. Greenwood, Guylaine Ferland, Pierrette Gaudreau, Sylvie Belleville, Danielle Laurin, Nicole D. Anderson, Marie-Jeanne Kergoat, Jose A. Morais, Nancy Presse, Alexandra J. Fiocco

**Affiliations:** 1 Department of Psychology, Ryerson University, Toronto, ON Canada; 2 PERFORM Centre, Concordia University, Montréal, QC Canada; 3 Rotman Research Institute, Baycrest Health Sciences, North York, ON Canada; 4 Department of Nutritional Sciences, University of Toronto, Toronto, ON Canada; 5 Department of Nutrition, Université de Montréal, Montréal, QC Canada; 6 Department of Medicine and Centre hospitalier de l’Université de Montréal (CHUM), Université de Montréal, Montréal, QC Canada; 7 Centre de Recherche de l’Institut Universitaire de Gériatrie de Montréal (CRIUGM), Montréal, QC Canada; 8 Centre d’excellence sur le vieillissement de Québec, Université Laval, Québec, QC Canada; 9 Department of Psychology, University of Toronto, Toronto, ON Canada; 10 Department of Psychiatry, University of Toronto, Toronto, ON Canada; 11 Division of Geriatric Medicine, McGill University, Montréal, QC Canada; 12 Faculty of Medicine and Health Sciences, Université de Sherbrooke, Sherbrooke, QC Canada; 13 Research Center on Aging, CIUSSS-de-l’Estrie-CHUS, Sherbrooke, QC Canada

**Keywords:** Older adults, Dietary patterns, Prudent diet, Western diet, Cognitive function, Sex differences

## Abstract

**Background:**

Consumption of a prudent dietary pattern rich in healthy nutrients is associated with enhanced cognitive performance in older adulthood, while a Western dietary pattern low in healthy nutrients is associated with poor age-related cognitive function. Sex differences exist in dietary intake among older adults; however, there is a paucity of research examining the relationship between sex-specific dietary patterns and cognitive function in later life.

**Methods:**

The current study aimed to investigate sex differences in the relationship between sex-specific dietary pattern adherence and global cognitive function at baseline and over a 3-year follow-up in 1268 community-dwelling older adults (*M*_*age*_ = 74 years, *n* = 664 women, *n* = 612 men) from the Quebec Longitudinal Study on Nutrition and Successful Aging (NuAge). A 78-item Food Frequency Questionnaire was used to estimate dietary intake over the previous year. Sex-specific dietary pattern scores were derived using principal component analysis. Global cognition was assessed using the Modified Mini-Mental State Examination (3MS).

**Results:**

Adjusted linear mixed effects models indicated that a healthy, prudent dietary pattern was not associated with baseline cognitive performance in men or women. No relationship was found between Western dietary pattern adherence and baseline cognitive function in women. Among men, adherence to an unhealthy, Western dietary pattern was associated with poorer baseline cognitive function (*β* = − 0.652, *p* = 0.02, 95% CI [− 1.22, − 0.65]). No association was found between prudent or Western dietary patterns and cognitive change over time in men or women.

**Conclusions:**

These findings highlight the importance of conducting sex-based analyses in aging research and suggest that the relationship between dietary pattern adherence and cognitive function in late life may be sex-dependent.

## Background

In the context of an aging population, it is important to identify effective prevention strategies that may slow cognitive deterioration in late life. Adherence to a healthy dietary pattern is emerging as a promising lifestyle behaviour that may protect against age-related cognitive decline [1]. More specifically, diets high in nutrients such as B-vitamins, monounsaturated fatty acids (MUFA), polyunsaturated fatty acids (PUFA), and antioxidants (e.g.*,* beta-carotene, vitamin C) are reported to have neuroprotective properties involved in maintaining cognitive health in later adulthood [2–4]. In contrast, diets high in saturated fatty acids and simple carbohydrates are associated with poor age-related cognitive functioning [5].

In recent years, researchers have shifted focus from investigating specific nutrients or food products to evaluating patterns of dietary intake as a more holistic approach when examining the association between nutrition and cognition in later adulthood. Among the theory-based dietary patterns that have emerged over the last decade, the Mediterranean diet (MediDiet) is the most widely investigated pattern. The MediDiet is characterized by high intake of vegetables, legumes, fruits and nuts, unrefined grains, and olive oil; moderate to high intake of fish/seafood; low to moderate intake of dairy products and meat and poultry; and moderate intake of ethanol primarily in the form of wine during meals [6]. Although mixed findings have been reported in various prospective studies [7–9], consumption of the MediDiet has been reportedly associated with higher global cognitive function and decreased risk for developing dementia [10–12]. These results are promising, but an important limitation with theory-based dietary patterns is that they do not capture the full complexity of the food matrix and may not reflect actual dietary intake.

An alternative approach to derive dietary patterns is the use of data-driven methods, which are not confined by theory, but rather reflect dietary intake within a given cohort. Studies using data-driven approaches have reported that adherence to an unhealthy “Western-type” dietary pattern (i.e.*,* high consumption of red meats and processed foods) is associated with poorer cognitive performance [13, 14] whereas adherence to a healthy “prudent” dietary pattern (i.e.*,* high consumption of fruits, vegetables, lean meats, nuts and seeds) is associated with better cognitive performance [13–18]. A recent systematic review of the literature on healthy dietary pattern adherence and cognitive function in older adulthood suggested an overall positive effect of adherence to a prudent dietary pattern on cognitive function [19]; however, a large amount of variation existed in the reported beneficial effects. A potential source of variation in the diet-cognition association may be biological sex.

Research suggests that reported dietary intake differs between men and women [20]. For example, women are less likely than men to report consuming an unhealthy Western-like dietary pattern [14]. Rather, women are more likely than men to report consumption of a healthy, prudent dietary pattern [13–17]. Although sex is usually controlled for in cohort studies examining the relationship between dietary intake and cognitive function in aging, sex differences in dietary intake, whether characterized as healthy or unhealthy, suggest that the food items within dietary patterns may differ among older men and women.

To date, one study has investigated sex differences in the diet-cognition relationship in later adulthood, using cross-sectional data from the Three-City study [21]. Using sex-stratified cluster analysis of a food frequency questionnaire, both common and sex-specific clusters were derived. Common clusters included “small eaters”, “biscuits and snacking”, and a “healthy” cluster. Sex-specific clusters included a “charcuterie, meat and alcohol” cluster and a “pasta eater” cluster for men; and a “charcuterie, starchy foods” cluster and a “pizza, sandwich” cluster for women. The healthy cluster included slight sex-specific variations in food components, including intake of fruits and vegetables in women and intake of pasta, fish and seafood in men. Despite variations in patterns of dietary intake between men and women, only the healthy cluster was negatively associated with errors on the Mini-Mental State Examination (MMSE) in both sexes. Limitations of this study important to highlight include failure to control for energy intake, and derivation of dietary patterns that are difficult to interpret from a public health perspective as they do not reflect current guidelines on optimal eating patterns [22]. While no sex-specific effect was observed in the diet-cognition relationship, sex differences in dietary components warrant further investigation using sex-based dietary patterns.

To this end, the objective of the current study was to examine the relationship between data-driven, sex-based patterns of dietary intake and global cognitive function in older adult men and women at baseline and over a 3-year follow-up period. Based on previous literature, it was hypothesized that components of healthy and unhealthy dietary patterns would differ between sexes. It was also hypothesized that dietary pattern intake would differentially associate with baseline global cognitive function and cognitive change in men and women.

## Methods

### Study cohort

The current sample included older adults from the Quebec Longitudinal Study on Nutrition and Successful Aging (NuAge), a prospective study of cognitively intact and functionally independent older adults aged 67 to 84 years at baseline, recruited from the Quebec Medicare database in 2003–2005. For further details on this population-based cohort study, see Gaudreau et al. [23]. Briefly, participants were globally healthy and did not present evidence of cognitive impairment at baseline, as assessed by the Modified Mini-Mental Examination (3MS score > 79), or disabilities in activities of daily living. Following provision of consent, all participants were tested at Research Centers of the Institut Universitaire de Gériatrie de Montréal or the Institut Universitaire de Gériatrie de Sherbrooke. The current study is based on the first 3 years of follow-up (mean follow-up = 3.0 ± 0.2 years).

From the original 1793 NuAge participants, 1754 (98%) agreed to have their data integrated into the NuAge Database and Biobank for future studies. For the purpose of the current study, participants were excluded a priori from the current analyses if they reported cerebral vascular disease, Parkinson’s disease, epilepsy, and/or muscular dystrophy due to their potentially negative impact on brain health. Participants were also excluded if they consumed < 800 or > 5000 kcal (kcal) per day or had 10% or more responses missing as assessed by a semi-quantitative food frequency questionnaire (FFQ; see details below). The final analytical sample included 1276 older adults at baseline (2003/2005), 1140 at 1-year follow-up (2005/2006), 1121 at 2-year follow-up (2006/2007), and 1045 at 3-year follow-up (2007/2008).

### Measures

#### Demographic and health-related characteristics

The following non-dietary variables were collected during a structured, computer-assisted interview at baseline: age, sex, education, body mass index [BMI; measured weight (kg)/measured height (m^2^)], seated systolic (SBP) and diastolic (DBP) blood pressure, presence of hypertension (self-report diagnosis, use of antihypertensive medication, SBP > 140 mmHg or DBP > 90 mmHg), and type-2 diabetes (self-report diagnosis, use of insulin or oral hypoglycemic medication, fasting serum glucose concentration ≥ 7.0 mmol/L). Women were also asked whether they currently or have ever received estrogen treatment.

#### Dietary intake

Dietary intake was measured at baseline by a validated, semi-quantitative food frequency questionnaire (FFQ) which estimates usual intake of sample portions of 78 different food items over the previous 12 months on a 6-point scale from ‘never or rarely’ to ‘2 times or more per day’ [24]. Participants were also asked to indicate whether their usual portion size for each food item was smaller, similar to, or larger than the sample portion size illustrated in the FFQ. The FFQ was developed, tested, and validated in English and French speaking adults living in Quebec, Canada [24, 25].

#### Cognition

The Modified Mini-Mental State Examination (3MS) was administered at baseline and annually for 3 years to evaluate global cognitive function and detect possible cognitive impairment. The 3MS is an extended version of the Mini Mental State Examination [26] assessing five areas of global cognitive function: orientation, registration, attention and calculation, recall, and language. Scores on the 3MS range from 0 to 100, with higher scores indicating better global cognitive function.

### Statistical analyses

Sex-specific dietary patterns were derived using varimax rotated principal component analysis (PCA) with the FFQ food items. Prior to deriving dietary patterns, the frequency of consumption of each food item was multiplied by 0.5, 1, or 1.5 if the portion size was reported to be smaller, similar, or larger than that presented in the FFQ, respectively. The number of factors retained in the PCA was determined by considering eigenvalues > 1, the scree plot, and interpretability of the resulting patterns. Factor loadings with an absolute value > 0.209 for men and > 0.201 for women were considered significant contributors to the interpretation of the factors as per guidelines by Norman and Streiner [27]. These cut-off scores represent the critical value for a correlation based on the sample size at baseline (women: *n* = 664; men: *n* = 612). Dietary pattern scores for each participant were calculated by converting the average score for each category belonging to the given pattern to a z-score.

Sex differences in baseline characteristics were calculated using *t*-tests for continuous variables, and *χ*^*2*^ for categorical variables. Associations between sex-specific dietary patterns, baseline cognitive function, and cognitive change over 3 years were determined by linear mixed effects models using the “nlme” [28] and “interaction” [29] statistical packages on R [30], with an unstructured covariance structure. Time was coded as a continuous variable expressed as years since baseline for each follow-up period. The parameter estimates for Diet represented the association between dietary pattern adherence and baseline cognitive performance. The parameter estimates for Diet × Time represented the association between dietary pattern adherence and cognitive decline over the follow-up period (i.e.*,* slope). Positive estimates indicate that greater adherence to the dietary pattern is associated with less decline during follow-up. Dietary intake and covariates were entered as fixed effect factors and participants were specified as a random effects factor. All models were adjusted a priori for age, education (< 12 years, ≥ 12 years), BMI, diabetes, daily energy intake (kcal), and hypertension. All models were also adjusted a priori for past or current estrogen use in women. Statistical significance for all analyses was set at *p* < 0.05.

## Results

### Participant characteristics

The majority of participants were Caucasian (98.7%). Participants ranged in age from 67 to 84 years with 44.7% aged 75 years and older. Mean scores on the 3MS at baseline and at each subsequent annual visit were: 94.5 ± 4.1, 93.8 ± 5.1, 93.1 ± 5.7, and 93.2 ± 6.3 for women, and 93.5 ± 4.5, 91.9 ± 6.1, 91.9 ± 6.5, and 91.6 ± 6.2 for men. On average, women scored higher than men on the 3MS at baseline (*p* < 0.001), were more likely to have hypertension (*p* = 0.003) and less likely to have diabetes (*p* < 0.001). No sex differences were found for age, education, or BMI. Participant demographic and health-related characteristics, including cognitive performance on the 3MS, for the total sample and stratified by sex are shown in Table 1.

**Table 1:** Participant Demographic and Health-Related Characteristics

	Total (*n* = 1276)	Women (*n* = 664)	Men (*n* = 612)	*p*-value
Age (years)	74.16 ± 4.16	74.23 ± 4.23	74.08 ± 4.08	0.53
Race (% Caucasian)	98.7	98.9	98.5	0.68
Education (% < 12 years)	50.1	52.0	48.2	0.20
BMI (kg/m^2^)	27.80 ± 4.66	27.61 ± 5.21	28.00 ± 3.99	0.14
Hypertension (%)	47.0	51.1	42.6	**0.003**
Diabetes (%)	10.8	7.8	14.1	**< 0.001**
Baseline 3MS score	94.04 ± 4.32	94.53 ± 4.14	93.50 ± 4.45	**< 0.001**
Energy intake (kcal)	1818 ± 527	1702 ± 458	1943 ± 566	**< 0.001**
Current or past estrogen use (%)	–	56.1	–	–
Prudent diet pattern (min. – max.) ^a^	–	−1.96 – 7.12	−1.85 – 6.43	–
Western diet pattern (min. – max.) ^a^	–	−3.10 – 3.86	−2.50 – 4.08	–

*p*-value derived by *t*-test for continuous variables and *χ*^*2*^ for categorical variables. Data presented as mean ± standard deviation unless otherwise specified

^a^Adherence to prudent and Western dietary pattern presented as z-scores derived from principal component analysis

*BMI* body mass index, *3MS* Modified Mini-Mental State Examination

### Sex-specific dietary patterns

In men, two factors identified by PCA accounted for 5.8 and 5.1% of the total variance. The first pattern was characterized by high consumption of fruits, vegetables, fatty fish, legumes, nuts and seeds, rice and rice dishes, poultry, high-fiber cereals, tofu, and lower-fat dairy products, and was labelled the Prudent dietary pattern. The second dietary pattern was characterized by high consumption of red and processed meats, pork, potatoes, white bread, fried foods, butter, baked goods, sugary foods and drinks, high-fat dairy products, eggs and egg dishes, and salty snacks, and was termed the Western dietary pattern. A list of factor loadings for each food item are shown in Supplementary Table 1.

In women, two factors were identified by PCA, and accounted for 5.4 and 4.6% of the total variance. The first pattern, labelled the Prudent dietary pattern, was high in fruits, vegetables, fatty fish, poultry, rice and rice dishes, legumes, eggs and egg dishes, nuts and seeds, tofu, low-fat dairy products, and cheese. The second pattern, labelled the Western dietary pattern, was characterized by high consumption of red and processed meats, pork, white bread, potatoes, fried foods, butter, pasta, baked goods, sugary foods and drinks, high-fat dairy products, and salty snacks. Factor loadings for each food item are shown in Supplementary Table 2.

### Dietary pattern adherence and cognitive function

Unadjusted linear mixed effects models revealed that, in men, higher adherence to the prudent dietary pattern was associated with better baseline cognitive performance (*β* = 0.534, *p* = 0.04); whereas greater adherence to the Western dietary patterns was associated with poorer baseline cognitive performance (*β* = − 0.713, *p* = 0.005). In women, higher adherence to the prudent dietary pattern was also associated with better baseline cognitive performance (*β* = 0.654, *p* = 0.004), while the Western dietary pattern was not associated with baseline cognition (*β* = − 0.368, *p* = 0.11).

Adjustment of the linear mixed effects models for covariates in men did not significantly change the association between Western dietary pattern adherence and baseline cognition (*β* = − 0.652, *p* = 0.02); however, the association between prudent dietary pattern adherence and baseline cognition was no longer statistically significant following adjustment for covariates (*β* = 0.313, *p* = 0.22). In women, the association between prudent dietary pattern adherence and baseline cognitive function was no longer statistically significant following adjustment for covariates (*β* = 0.403, *p* = 0.12). Linear mixed effects models showed significant decline in 3MS performance over time in men (*β* = − 0.63, *p* < 0.001) and women (*β* = − 0.48, *p* < 0.001), which remained significant after controlling for covariates. However, unadjusted and adjusted models revealed that the slope of cognitive decline did not change as a function of degree of adherence to the prudent or Western dietary patterns in men and women (*p*s > 0.10). See Table 2 for all model details.

**Table 2:** Association Between Sex-specific Dietary Patterns, Baseline Cognition, and Cognitive Change Over 3 Years

	Unadjusted			Adjusted ^a^		
	*β*	*SE*	*p*-value	*β*	*SE*	*p*-value
Women ^b^						
Time	−0.47	0.06	**< 0.001**	− 0.48	0.06	**< 0.001**
Prudent Diet	0.65	0.23	**0.004**	0.40	0.26	0.12
Prudent Diet × Time	−0.05	0.06	0.35	−0.03	0.06	0.60
Western Diet	−0.36	0.23	0.11	−0.31	0.27	0.26
Western Diet × Time	−0.06	0.06	0.26	−0.08	0.06	0.16
Men						
Time	−0.63	0.07	**< 0.001**	−0.64	0.07	**< 0.001**
Prudent Diet	0.53	0.26	**0.04**	0.31	0.26	0.22
Prudent Diet × Time	0.04	0.07	0.55	0.04	0.07	0.54
Western Diet	−0.71	0.25	**0.005**	−0.65	0.29	**0.02**
Western Diet × Time	−0.08	0.07	0.23	−0.08	0.07	0.21

^a^ Adjusted for age, education (< 12, ≥12 years), energy intake in kcal, BMI, diabetes, and hypertension; ^b^ Additionally adjusted for estrogen past or current estrogen use in women

## Discussion

The role of lifestyle behaviors in the maintenance of cognitive function in later adulthood has received increasing attention. Among the various lifestyle behaviors under investigation, patterns of dietary intake have shown promising results in modulating cognitive function among older adults. In more recent years, the importance of sex and gender-based analyses has come forth as an important line of investigation given differential biological and cultural exposures that may influence lifestyle behaviors and cognitive functioning [31]. The objective of this study was to determine if sex-specific dietary patterns were associated with global cognitive function in non-demented, community-dwelling older adults at baseline and over a 3-year follow-up period. Dietary patterns characterizing a prudent and Western diet were derived for both men and women. Overall, the study findings suggest that men may be more sensitive to the effects of an unhealthy Western dietary pattern, relative to women. Further, adherence to a prudent dietary pattern may not be sensitive enough to independently associate with cognitive health [18]. Finally, results showed that null association between dietary patterns and global cognitive change was comparable in men and women.

In the current study, PCA analysis identified two dietary patterns that are consistent with previous research using a data-driven approach to derive dietary patterns. Shakersain et al. [14] and Gardener et al. [32] reported similar Western and prudent dietary patterns using PCA analysis in older adults, with only slight variations in exact food items. Importantly, although it is not possible to statistically compare sex-based PCA-derived patterns, the current study suggests that similar dietary patterns were evident in men and women. This observation was unexpected given previous reports of sex differences in dietary pattern adherence [14]. Similarities in dietary food matrices within each pattern across sex suggests that sex differences in the diet-cognition relationship may not be due to different foods consumed, but rather to differences between men and women in the effectiveness of diet to impact cognitive function in late life.

Sex-based differences in biophysiological sensitivity to dietary intake may further explain the observed association between Western dietary pattern adherence and baseline cognitive function in men. For example, high levels of triglycerides have been found to associate with cognitive impairment, defined by a score ≤ 17 on the MMSE among men but not women [33]. Furthermore, investigating the association between homocysteine, a metabolic marker that is commonly associated with high adherence to a Western diet and poorer cognitive function, Gao et al. [34] reported that a higher level of plasma homocysteine was associated with poorer global cognitive function among men, but not women. These studies, however, have not controlled for possible confounding variables such as kidney function and medication use, warranting the need for future study.

Although the aforementioned sexual dimorphic associations may help shed light on the current study findings, it is important to recognize inconsistencies in the literature that examine the association between peripheral biomarkers and cognitive function [35]. For example, sex-specific associations between lipids and cognitive function have been mixed, depending on the lipid and the specific cognitive domain under investigation [36, 37], warranting a need for future studies to examine dietary patterns and multiple cognitive domains. Moreover, biological mechanisms through which dietary patterns affect cognitive function are likely due to an integrative effect of multiple biological systems and physiological pathways such as inflammatory markers, lipid profile, and vitamin intake [35]. Genetic polymorphisms may also mediate the relationship between dietary pattern adherence and cognitive function through the regulation of systemic biomarkers [38–40]. For example, genetic polymorphism of the apolipoprotein E gene (APOE) is associated with cognitive function and contributes to the regulation of circulating lipids [39–41]. Consequently, there is a need to comprehensively investigate biological and genetic mediators of sex-specific associations between dietary patterns and cognitive function in late life.

A null association between prudent dietary pattern adherence and baseline global cognitive function was found in both men and women. This is inconsistent with a previous study by Shakersain et al. [14], which reported that the highest adherence to a prudent dietary pattern was associated with less global cognitive decline over 6 years relative to the lowest adherence among 2223 dementia-free older adults. Contrasting results between studies may stem from differences in sample size, which may influence statistical power, and/or from the statistical approach of dividing a continuous dietary pattern variable into quintiles. Null associations in the current study may also be a result of the high-functioning cohort of participants who performed relatively well on the 3MS.

Despite the need for future investigation and replication, the current study findings are important as they contribute to the growing body of literature examining the association between dietary pattern intake and cognitive function in later life using a data-driven approach. Furthermore, given the paucity of sex-based analyses in this area of research, the current study supports the need for sex-based analyses to better understand the association between dietary intake and cognitive function, without the assumption that the benefits of a particular dietary pattern will contribute to the health of men and women equally.

Study limitations must be acknowledged, which may have overestimated or underestimated the observed associations. First, the cohort under investigation consisted of healthy independent living older adults. As such, findings cannot be generalized to persons with existing neurological impairments, including Parkinson’s disease and epilepsy, as cohort participants presenting with these disorders were excluded from the present study analyses. As a whole, the study sample displayed small age-related changes in global cognitive trajectory over the three-year follow-up period and did not display clinically significant rates of decline, defined as a 5-point decline per year on the 3MS [42]. Indeed, 4.4% of men and 6.3% of women declined by 5 points or more on the 3MS over the 3-year follow-up. Accordingly, the association between dietary pattern adherence and cognitive change over time may have been underestimated due to a ceiling effect in cognitive performance. Longer follow-up assessments may be required to capture the effects of dietary pattern adherence on cognitive trajectory among high functioning older adults. Indeed, previous studies that have reported a relationship between data-driven dietary pattern adherence and change in cognitive function over time followed participants for 6 to 13 years [14, 15, 17]. Furthermore, assessment of global cognition using the 3MS may have underestimated the relationship between diet and cognitive function, as variability in performance was relatively low in the current sample. Future studies may benefit from assessing specific cognitive domains such as executive function and episodic memory, which may be more sensitive to age-related changes in a healthy, high-functioning sample.

Another study limitation to acknowledge is the timing of dietary assessment. Although the current study was based on a longitudinal cohort, dietary intake was only assessed at baseline, which does not capture potential changes in diet over the 3-year follow-up period. This lack of information precludes analyses that may elucidate how changes in dietary patterns over time may associate with changes in cognitive function. Further, considering that the study sample was primarily Caucasian, the reported dietary patterns may not hold true for more ethnically diverse populations, minimizing the ability to generalize current findings beyond the study sample. Along a similar vein, the current relevance of study findings may be compromised due changes in dietary tends over time. Specifically, dietary intake was assessed between 2003 and 2005; however, adherence to prudent-like dietary patterns among older adults has likely increased over the last two decades [43, 44].

Finally, differences across studies investigating dietary patterns using a data-driven, a posteriori approach limits inter-study comparisons. It is not possible to statistically compare levels of prudent and Western dietary adherence between men and women using data-driven methods. However, data-driven approaches to dietary pattern analysis provide insight into the dietary behaviour of the study participants without imposing predetermined guidelines for healthy eating behaviour.

Although the relationship between Western dietary pattern adherence and baseline cognitive function among men was statistically significant, the proportion of explained variance in the models was relatively low, raising the possibility of residual confounding. Thus, it is important to consider additional lifestyle behaviours that may be impacting the results. Indeed, the influence of multiple unhealthy lifestyle behaviours in addition to poor dietary intake, such as smoking, lack of physical activity, and medical comorbidities other than diabetes and hypertension, often cluster and may interact with one another to exert effects on cognitive functioning in late life [45, 46]. As such, future research should consider additional lifestyle behaviours when assessing the sex-dependent relationship between dietary pattern intake and cognitive function in later life.

## Conclusion

Findings from this study contribute to the growing body of research dedicated to identifying modifiable lifestyle behaviours that facilitate healthy cognitive aging. Further, the current study highlights the importance of considering the differential association between lifestyle factors and cognitive health as a function of sex. This serves as a stepping-stone for future research to investigate the effects of sex on the diet-cognition relationship, and to explore the biological mechanisms that underlie this association.

## Supplementary information

**Additional file 1: Supplementary Table 1.** Factor loadings for dietary patterns in men. *Note.* Loadings (>|0.209|) represent correlation of an FFQ item with dietary pattern score.

**Additional file 2: Supplementary Table 2.** Factor loadings for dietary patterns in women. *Note.* Loadings (>|0.201|) represent correlation of an FFQ item with dietary pattern score.

## Data Availability

NuAge data access for this project was approved by members of the NuAge Steering Committee in March 2015 and the NuAge dataset was obtained thereafter. The NuAge datasets used in the current study are available by submitting an access request to the NuAge Database and Biobank Steering Committee (NuAge-cdrv@usherbrooke.ca).

## Abbreviations

3MS: Modified mini-mental state examination
APOE: Apolipoprotein-E gene
BMI: Body mass index
DBP: Diastolic blood pressure
FFQ: Food frequency questionnaire
MediDiet: Mediterranean diet
MMSE: Mini-mental state examination
MUFA: Monounsaturated fatty acid
NuAge: Quebec Longitudinal Study on Nutrition and Successful Aging
PCA: Principal component analysis
PUFA: Polyunsaturated fatty acid
SBP: Systolic blood pressure
