# Supplementary material for: Sex differences in the relationship between dietary pattern adherence and cognitive function among older adults: findings from the NuAge study
Source: Nutr J. 2020 Jun 20;19:58. doi: 10.1186/s12937-020-00575-3 (PMC7306140; doi:10.1186/s12937-020-00575-3)
Supplement: Supplementary file 2 — Additional file 2: Supplementary Table 2. Factor loadings for dietary patterns in women. Note. Loadings (>|0.201|) represent correlation of an FFQ item with dietary pattern score. [file 12937_2020_575_MOESM2_ESM.docx]

*Supplementary Table 2.* Factor loadings for dietary patterns in women.

| Food item | Prudent diet | Western diet |
| --- | --- | --- |
| Green, leafy vegetables (summer + rest of the year) | 0.651 | - |
| Red, green, yellow, sweet peppers | 0.580 | - |
| Cruciferous vegetables | 0.530 | - |
| Other vegetables | 0.522 | - |
| Other fruits | 0.421 | - |
| Tomatoes (summer + rest of the year) | 0.411 | - |
| Berries | 0.408 | - |
| Salmon, trout, sardines, herring, tuna | 0.396 | - |
| Salad dressings, mayonnaise dips | 0.395 | - |
| Carrots | 0.379 | - |
| Yogurt | 0.378 | - |
| Green/yellow beans, green peas, corn | 0.331 | - |
| Seafood | 0.328 | - |
| Nuts, peanuts, other seeds | 0.312 | - |
| Beans, peas, lentils, hummus, beans with pork | 0.290 | - |
| Melons | 0.286 | - |
| Rice, rice noodles, couscous | 0.279 | - |
| Eggs, omelettes, quiches | 0.270 | - |
| Citrus fruit | 0.250 | - |
| Bananas | 0.232 | - |
| Tofu, foods with soya or vegetable protein | 0.229 | -0.201 |
| Other fish | 0.226 | - |
| Poultry | 0.217 | - |
| Cheeses | 0.211 | - |
| Tomato or vegetable soups | 0.202 | 0.202 |
| Boiled, mashed, baked potatoes | - | 0.515 |
| Beef | - | 0.485 |
| Sauces (brown, white, BBQ, gravy) | - | 0.455 |
| Baked goods (cakes, pies, donuts, pastries) | - | 0.443 |
| French fries or pan-fried potatoes | - | 0.384 |
| Commercial sliced white bread | -0.225 | 0.382 |
| Ice cream, frozen yogurt | - | 0.367 |
| Sausages, hot dogs | - | 0.360 |
| Regular soft drinks | - | 0.340 |
| Ham, cold cuts, smoked meat, bacon | - | 0.336 |
| Pork | - | 0.318 |
| Milk or cream in coffee/tea | - | 0.304 |
| Coffee/tea | - | 0.275 |
| Salty snacks (chips, salted crackers, popcorn, pretzels) | - | 0.270 |
| Sugar in coffee/tea | - | 0.269 |
| Margarine on bread or cooked vegetables | - | 0.268 |
| Fruit drinks with added sugar | - | 0.263 |
| Pasta with tomato sauce | - | 0.259 |
| Pasta with cream sauce | - | 0.251 |
| Milk-based desserts, puddings | - | 0.233 |
| Pizza | - | 0.233 |
| Liver, other organ meats | 0.228 | 0.232 |
| Sugar added to cereal | - | 0.223 |
| Butter on bread or cooked vegetables | - | 0.222 |
| Jam, honey, sweet spreads, maple products | - | 0.218 |
| Other soups | - | 0.202 |
| Candies, chocolate | - | - |
| Soya drinks | - | - |
| Whole-wheat bread | - | - |
| High fiber breakfast cereals | - | - |
| Other cereals | - | - |
| Other whole-wheat breads | - | - |
| Other white breads | - | - |
| Cookies | - | - |
| Peanut butter | - | - |
| Other meats | - | - |
| Vegetable juices | - | - |
| Apples, pears | - | - |
| Muffins | - | - |
| Granola bars | - | - |
| Sugar-free fruit drinks | - | - |
| Diet soft drinks | - | - |
| Whole milk | - | - |
| 1% milk, 2% milk | - | - |
| Skim milk | - | - |
| Beer | - | - |
| Wine | - | - |
| Spirits | - | - |
| Sunflower seeds | - | - |

*Note.* Loadings (>|0.201|) represent correlation of an FFQ item with dietary pattern score
